# Two tropical seagrass species show differing indicators of resistance to a marine heatwave

**DOI:** 10.1002/ece3.10304

**Published:** 2023-07-14

**Authors:** Alissa V. Bass, Laura J. Falkenberg

**Affiliations:** ^1^ Simon F.S. Li Marine Science Laboratory The Chinese University of Hong Kong Sha Tin New Territories Hong Kong SAR

**Keywords:** climate change, ecophysiology, growth, *Halophila beccarii*, *Halophila ovalis*

## Abstract

Marine heatwaves (MHWs) are a growing threat to marine species globally, including economically and ecologically important foundation species, such as seagrasses. Seagrasses in tropical regions may already be near their thermal maxima, and, therefore, particularly susceptible to increases in temperature, such as from MHWs. Here, we conducted a 10‐day MHW experiment (control +4°C) to determine the effects of such events on the two tropical seagrasses *Halophila beccarii* and *Halophila ovalis*. We found that both species were largely resistant to the MHW, however, there were differences between the species' responses. For *H. beccarii*, the surface area of existing leaves was smaller under MHW conditions, yet a substantial increase in the number of new leaves under the MHW indicated its tolerance to—or even increased performance under—the MHW. While there was no direct effect of the MHW on *H. ovalis*, this species saw less epiphyte biomass and percentage cover on its leaves under the MHW. While a lower epiphyte cover can potentially increase the health and ecophysiological performance of the seagrass, the change of epiphytes can lead to bottom‐up trophic implications via the influence on mesograzer feeding. Together, the results of this study demonstrate the species‐specific responses of seagrasses of the same genus to a warming event. With the current global decline of seagrasses, our results are encouraging for these important habitat formers as we show that anomalous warming events may not necessarily lead to ecosystem collapse.

## Graphical abstract

Here, we conducted a 10‐day marine heatwave (MHW) experiment (control +4°C) to determine the effects of such events on the two tropical seagrasses *Halophila beccarii* and *Halophila ovalis*. We found that both species were resistant to the MHW, however, there were differences between the species' responses. With the current global decline of seagrasses, our results are encouraging for these important habitat formers as we show that anomalous warming events may not necessarily lead to ecosystem collapse.

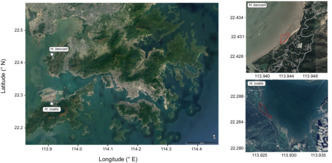

## 1 INTRODUCTION

Seagrasses are important marine foundation species, occurring in most shallow, sheltered, soft sediment coastlines and estuaries of the world (Den Hartog, 1970; McKenzie et al., 2020; Short et al., 2007). Due to the many ecosystem functions and services seagrasses provide, they are one of the most valuable ecosystems globally (Costanza et al., 2014). For example, seagrasses are responsible for approximately 15% of carbon storage in the ocean (Duarte & Cebrián, 1996; Duarte & Chiscano, 1999), despite only covering approximately 0.2% of the Earth's surface. Furthermore, seagrasses provide important trophic links with other ecosystems (Green & Short, 2003; Heck et al., 2008), exporting on average almost 25% of their net production to adjacent ecosystems (Duarte & Cebrián, 1996). Other services delivered by seagrass beds include provision of food, oxygen, sediment stabilisation, improvement of water transparency, wave attenuation and shoreline protection (Barbier et al., 2011; Duarte & Cebrián, 1996; Nordlund et al., 2016).

Climate change and other anthropogenic impacts can drive large‐scale effects on seagrasses, with these meadows disappearing at a rapid rate (Orth et al., 2006; Short & Neckles, 1999; Waycott et al., 2009). Over recent decades loss of seagrass has been reported to be occurring globally at a rate of approximately 110 km^2^ year^−1^, making them one of the most threatened ecosystems in the world (Waycott et al., 2009). Specifically, seagrass loss has been recorded in the Americas (Short et al., 2006), the Mediterranean (Marbà et al., 2014), Asia (Unsworth et al., 2018) and Australia (Kendrick et al., 2019). Additionally, further loss, distribution shifts and altered ecophysiological performance are expected in the future as anthropogenic impacts increase (Chefaoui et al., 2018; Nguyen et al., 2000; Repolho et al., 2017).

One climate change factor that can have, and is already having, a prominent effect on seagrasses is marine heatwaves (Smale et al., 2019; Smith et al., 2023). Marine heatwaves (MHWs) are defined as five or more days of anomalous sea surface temperature above the regional 90th percentile (Hobday et al., 2016). These MHW events are increasing in intensity, frequency and duration and are predicted to continue doing so in the future (Oliver et al., 2018, 2019). MHWs can have important ecological effects and have been shown to degrade seagrass in temperate regions (Arias‐Ortiz et al., 2018; Marbà & Duarte, 2010; Serrano et al., 2021; Strydom et al., 2020; Thomson et al., 2015). Tropical seagrass species have important interactions with mangroves and corals (Green & Short, 2003) and while seagrass studies in the tropics were previously lacking (Unsworth et al., 2018), there has been an increase in research considering their responses to anomalous warming events (Rasmusson et al., 2021; Strydom et al., 2020; Szitenberg et al., 2022; Thomson et al., 2015). It is important to study seagrasses in these regions given that the bulk of extant seagrass meadows is found on the coastline of developing tropical countries (Duarte, 2002), which are experiencing the greatest rate of environmental degradation and are projected to continue to do so in the future.

The loss of seagrass meadows can have repercussions for many trophic levels and on the whole ecosystem functioning. That is, seagrasses are an important food source for macro herbivores (Bjorndal, 1980; Marsh et al., 1982) and mesograzers (Ebrahim et al., 2014; Klumpp et al., 1992), as well as a blue carbon sink (Gullström et al., 2018). In tropical systems extreme climatic events can have severe effects on traits (e.g. survival, abundance) of habitat‐forming species including seagrasses, with prolonged impacts for slow‐growing species than their faster‐growing counterparts (Babcock et al., 2019).

The tropical seagrasses *Halophila beccarii* Aschers., and *Halophila ovalis* (R. Br.) Hook. *f*. belong to the family Hydrocharitaceae, Order Alismatales. *Halophila* seagrasses have a tropical and warm temperate distribution, and many are endemic with limited distributions (Short et al., 2007). For the two species used in this study, *H. ovalis* has a wide subtropical and tropical distribution, ranging from the west coast of Africa to South Asia, South‐East Asia and Australia. *H. beccarii* also has a wide, yet patchy, distribution in the tropical Indo‐Pacific (Green & Short, 2003). In Hong Kong, these species are found on the low intertidal and shallow subtidal on mudflats, and are associated with mangroves and also with seaweed in spring (Morton & Morton, 1983, personal observation). Compared to slow‐growing and larger seagrasses in these regions and globally, both *H. ovalis* and *H. beccarii* are small, quickly colonising and opportunistic. Consequently, they can potentially recover quickly after habitat disturbance relative to other seagrasses. Here, we performed a 10‐day marine heatwave experiment and considered the responses of *H. beccarii* and *H. ovalis* to MHW events which we hypothesised they would be largely resistant to, with specific responses dependent on the growth and morphological traits considered. The aims of this experiment were, therefore, to: (i) investigate the vulnerability or resistance of two tropical seagrasses to a spring marine heatwave, (ii) identify the parameters of seagrass growth and morphology that are the most affected by a marine heatwave and (iii) consider if there is a difference in responses between seagrass species.

## 2 MATERIALS AND METHODS

### 2.1 Marine heatwave historical data

Daily Optimum Interpolation Sea Surface Temperature (OISST v2.1 from https://coastwatch.pfeg.noaa.gov/erddap/) data from the United States National Oceanic and Atmospheric Administration (NOAA) was used to support the heatwave temperature treatments for this experiment. SST temperature data was downloaded for pixels covering the longitudinal and latitudinal boundaries for Hong Kong SAR (113°48′ N, 22°10′ E–114°30′ N, 22°36′ E). Daily 0.25° OISST data within these boundaries were averaged before detecting MHWs using the ‘heatwaveR’ package (version 0.4.6; Schlegel & Smit, 2018). The almost 40‐year period from 1 January 1982 to 31 March 2022 was used to quantify: (a) the number of MHW events (as defined by Hobday et al., 2016), (b) the mean intensity and duration of each MHW and (c) the maximum intensity and duration MHW. From 1982 to 2022 (until 31st March) there were 92 MHWs in Hong Kong, with a mean intensity of 1.82°C and an average duration of 10.4 days. In May 2021 there was the highest intensity and longest‐lasting MHW on record for Hong Kong, which had an intensity of 5.83°C and duration of 42 days. As MHWs are projected to increase in intensity in the future (Oliver et al., 2019), the temperature treatments in the experiment were a control (the average spring SST for Hong Kong which is the season when this experiment was done—23°C) and a ‘severe’ category MHW treatment (Hobday et al., 2018) of +4°C above control temperature (to represent a likely intensity of near‐future MHWs—27°C).

### 2.2 Seagrass collection

**FIGURE 1:**
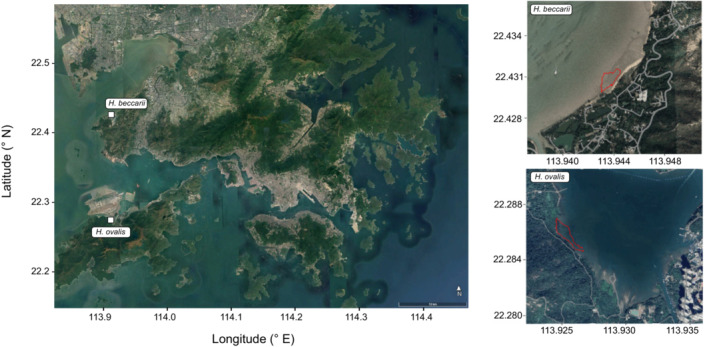
Locations of seagrass collection for *Halophila beccarii* and *Halophila ovalis*, and the extent of the seagrass in the two locations in March 2022 (maps from Google Earth Pro).

*Halophila beccarii* and *H. ovalis* were collected in March 2022 from Pak Nai, Deep Bay N22°25.8580′, E113°56.6609′ and Tung Chung Bay N22°17.0934′, E113°55.5946′, respectively (Figure 1). *Halophila beccarii* and its collection site are currently not under any kind of protection in Hong Kong, despite its vulnerable global status. Deep Bay, the site from which it was collected, is a proposed Marine Protected Area (MPA) in Hong Kong and is the habitat of the endangered horseshoe crabs *Tachypleus tridentatus* and *Carcinoscorpius rotundicauda* (Kwan et al., 2016). In contrast, Tung Chung Bay, the collection site for *H. ovalis*, is a Site of Special Scientific Interest (SSSI) and *H. ovalis* is listed in *Rare and Previous Plants of Hong Kong* (Hong Kong Herbarium), which protects the species from being collected without permission. Both seagrasses are found at the low intertidal and can be collected from the mudflat on extreme low tides. Using a handheld GPS (Garmin GPSMAP 66i), the area of the seagrass was recorded and five quadrats of 25 cm^2^ were haphazardly placed on the seagrass meadow and used to calculate shoot density. At Pak Nai, *H. beccarii* covered an area of approximately 11,709 m^2^ and shoot density varied between 240 and 640 shoots per 25 cm^2^. *Halophila ovalis* in Tung Chung Bay covered an area of approximately 15,928 m^2^ and the density was 300–400 shoots per 25 cm^2^. The seagrass was predominantly distributed in a large meadow, particularly toward the west of the site, however, there were also small patches of *H. ovalis* distributed irregularly throughout the mudflat. Seagrass and sediment adjacent to the meadows were collected using a trowel and placed into cool boxes. Spot measurements of temperature, pH and salinity were also taken during collection. For Tung Chung, the water temperature was 22.4°C, pH was 7.96 and salinity was 27 ppt, whereas at Pak Nai, water temperature, pH and salinity were 23.7°C, 8.24 and 24 ppt, respectively. Seagrass and sediment were transported to the Marine Science Laboratory at The Chinese University of Hong Kong, and the seagrass was placed in outdoor tanks and allowed to acclimate to ambient tank conditions for 2 days (as in Bass et al., 2023; Noisette & Hurd, 2018), with natural 12:12 h day/night light conditions. The weather was mostly overcast during the experiment and daytime irradiance levels outside varied between 250 and 1850 μmol m^−2^ s^−1^ depending on the weather conditions (MQ‐510 Full Spectrum Underwater Spectrum Meter; Apogee Instruments, Inc.). Following the acclimation period, individual ramets (seagrass shoots, attached to the rhizome and roots) were sorted, had initial measurements taken, were then planted into experimental tanks with the collected sediment (which was filtered using a metal sieve mesh size 4 mm—to remove biological material) with ambient air pumped into each tank and exposed to treatment temperatures.

### 2.3 Experimental design and response variable measurement

The experimental design consisted of subjecting collected seagrasses of each species (*H. beccarii* and *H. ovalis*) to either a control temperature (23°C) or a MHW temperature (+4°C, or 27°C). Five tanks were used per species and treatment combination (20 tanks in total). Within each 5 L tank, six ramets of the same species of a standardised size (detailed in the next paragraph) were planted into the filtered sediment which was 6 cm deep. The experiment ran for 10 days, with four additional days prior for ramping the water temperature up to experimental temperatures at a rate of 1°C per day (Bass et al., 2023; Minuti et al., 2021; Saha et al., 2020). To establish and maintain temperature treatments, seawater from the adjacent Tolo Channel was pumped into header tanks (one for each temperature treatment), which was connected to a chiller (HC‐1000B Chiller, Hailea®, China) for the control temperature treatment, or had water heaters inside (D‐839‐1000, Up‐aqua™, Taiwan) for the MHW treatment. This water was then pumped into each experimental tank, after which the water was able to flow out of the experimental tanks in a continuous flow‐through system (see Figure S1 for experimental setup schematic). Experimental temperatures were measured using temperature data loggers (iButton™, United Kingdom) placed into the experimental tanks (4–5 loggers per treatment) which were set to record every 10 min. These measurements revealed that the average experimental temperature for the treatments after the initial temperature ramping was 23.53°C (±0.02 SE) for the control and 27.48°C (±0.02 SE) for the MHW treatment.

Initial measurements were taken before planting the seagrass into the experimental tanks. This was done by first removing seagrass from its original sediment and rinsing with seawater to clear all remaining sediment and visible epiphytes and other epibiota. The seagrasses were standardised by cutting ramets to contain three shoots for *H. beccarii* or three shoot pairs for *H. ovalis*, as well as roots, and one apical meristem. Ramets were tagged using coloured cotton tied around the rhizome (adapted from Short & Duarte, 2001) in order to identify ramet and correct orientation for measurements at the end of the experiment. Ramets were then photographed before planting the ramets into the filtered sediment. Initial measurements were later recorded from these photographs, including rhizome length (horizontal and vertical rhizomes included), root length and leaf surface area using Fiji extension of Image J (Schindelin et al., 2012). Analysis of these initial measurements revealed that seagrasses of the same species had the same starting measurements across treatments, and, therefore, there was no initial bias.

After the 10‐day exposure to control or MHW water temperature, the seagrass was taken from their experimental tanks and rinsed gently with seawater to remove sediment. Ramets were identified and those which had completely disintegrated or were unidentifiable were categorised as ‘dead’. As mortality was low for both species, this response variable was excluded from formal statistical analysis. For those ramets that had survived, photographs were again taken and rhizome length, root length and the presence (and surface area) or loss of the original leaves were measured and analysed in ImageJ. Epiphyte percentage area was also recorded for the original leaves. For the new growth, that is, rhizome, roots, shoots and leaves that were not present at the start of the experiment, length measurements were taken of new rhizome growth, new root growth, the number of new leaves grown and also surface area measurements of the largest new leaf.

After taking the photographs for the above measurements, seagrass ramets were divided into sections for dry‐weight biomass quantification. These sections included epiphyte biomass (from three of the old leaves present at the initial measurement), leaf biomass (of the three old leaves and then of all leaves together), rhizome biomass and total root biomass. The sections of each ramet were placed in foil (pre‐weighed) before drying at 60°C for 2 days. Once measured, ramet total dry weight biomass was calculated by totalling the amounts of each individual section (with the exception of epiphyte biomass).

### 2.4 Statistical analyses

Measurements were then analysed in the statistical software *R* (version 4.2.0). The two species were analysed separately due to their inherent differences in morphology and growth strategies. For each response variable and each species, a mixed effects model was run with fixed factor ‘treatment’ and random effect of ‘tank number’ (i.e. each tank was treated as a replicate). A Levene's test was carried out for each species and response variable for test for homogeneity of data (Brown & Forsythe, 1974). Data were then transformed if needed (log or square root) to conform to normalisation and then the mixed effects model was performed using the package ‘lme4’ (Bates et al., 2015). Analysis of deviance using the Anova function was performed using the package ‘car’ (Fox & Weisberg, 2011) and *p*‐values of <.05 were used for significance. For zero‐inflated data, that is, epiphyte biomass for *H. beccarii*, a zero‐inflated model was fitted using the package ‘glmmTMB’ with a ‘beta’ transformation and a *p*‐value extracted using ANOVA, type III.

## 3 RESULTS

**FIGURE 2:**
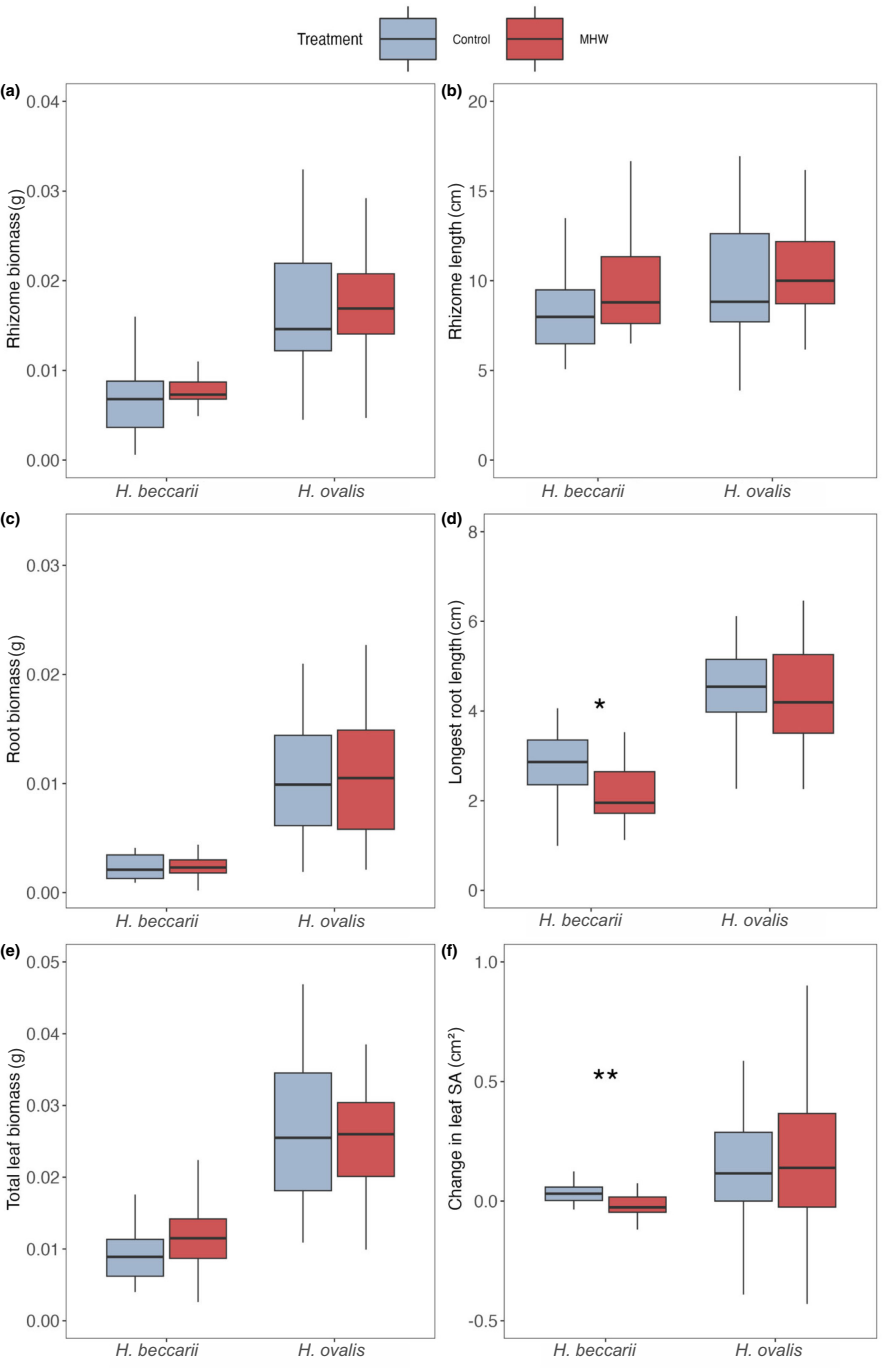
The dry weight biomass (DWB, g) and size (length or surface area, cm or cm^2^) of *Halophila beccarii* and *Halophila ovalis* exposed to marine heatwaves (control vs. heatwave) in terms of: (a) rhizome DWB, (b) rhizome length, (c) DWB of all roots on each ramet, (d) the length of the longest root, (e) total leaf DWB for each ramet and (f) average leaf surface area. Asterisks represent level of significance (*p < .05, **p < .01).

For both *H. beccarii* and *H. ovalis* there was no significant impact of MHW on mortality (of 30 ramets used for each species × treatment mortality observed for 5 vs. 7 ramets for *H. beccarii*, and 4 vs. 5 ramets for *H. ovalis* under control vs. MHW treatments, respectively), or on dry weight biomass of any of the ramet sections (rhizome, root, leaves; Figure 2a,c,e, respectively; Tables S1 and S2). Similarly, for *H. ovalis*, there was no significant effect of MHW on length or area for any of the seagrass segments (Figure 2b,d,f; Table S2). For *H. beccarii*, while rhizome biomass was similar across treatments, there was a trend toward greater rhizome length under MHW (10.10 cm vs. 8.35 cm, respectively; Figure 2b; Table S1). Root length of *H. beccarii*, however, was shorter under the MHW treatment, with an average of 2.17 cm compared to 2.81 cm at control (Figure 2d; Table S1; *F* = 6.4, *p* < .05). *H. beccarii* leaves present from the beginning of the experiment had a significantly lower surface area under the MHW treatment, compared to the control (averages of 0.117 cm^2^ vs. 0.317 cm^2^, respectively; Figure 2f; Table S1; *F* = 13.441, *p* < .01).

**FIGURE 3:**
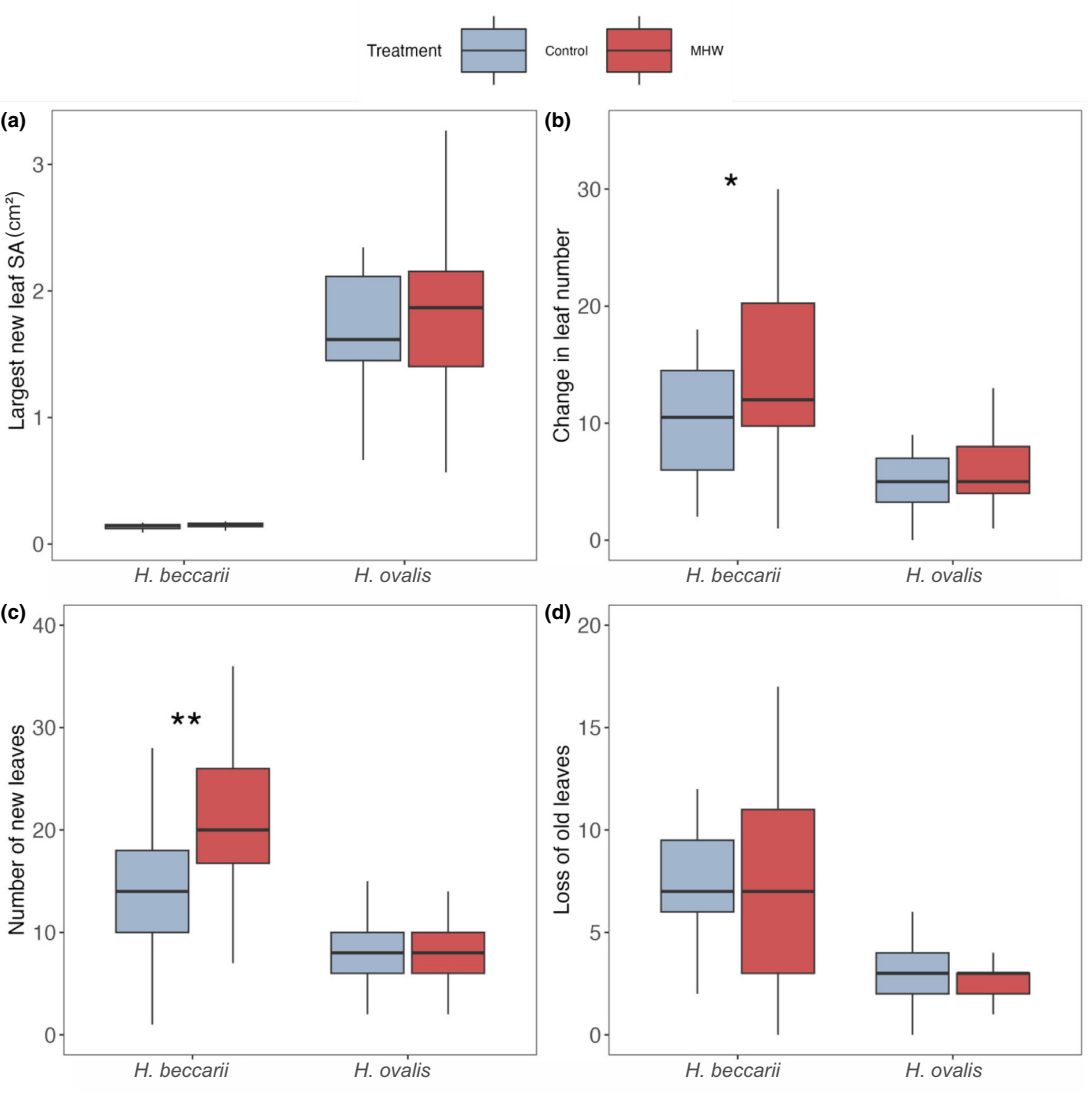
Leaf properties of *Halophila beccarii* and *Halophila ovalis* exposed to marine heatwaves (control vs. heatwave) in terms of: (a) largest new leaf surface area (cm^2^) on each ramet, (b) change in leaf number, (c) number of new leaves and (d) loss of old leaves present at the beginning of the experiment. Asterisks represent level of significance (*p < .05, **p < .01).

In terms of changes in the properties of leaves present, there was no difference in the size of new leaves grown on each ramet for either species in the control or MHW treatment (Figure 3a; Tables S1 and S2). The number of leaves at the end of the experiment compared to the start was, however, substantially boosted under MHW conditions for *H. beccarii* (averages of 14.20 leaves in MHW conditions compared to 6.13 under control conditions; Figure 3b; Table S1; *F* = 7.89, *p* < .05), which was associated with a greater production of new leaves (averages of 21.7 under MHW vs. 14.0 new leaves at control treatment; Figure 3c, Table S1, *F* = 6.14, *p* < .005). For *H. ovalis*, there was no difference in either change in leaf number or number of new leaves between the control and MHW treatment (Figure 3b,c; Table S2). There was also no difference in the number of leaves lost during the experiment between treatments for either species (Figure 3d; Tables S1 and S2).

**FIGURE 4:**
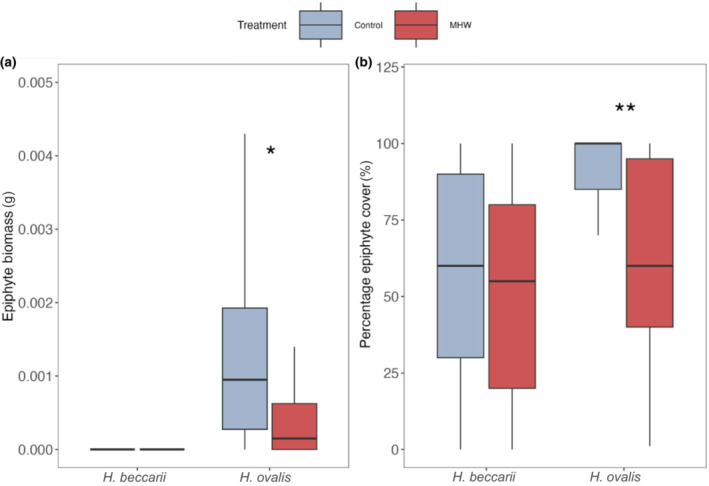
Epiphyte properties of *Halophila beccarii* and *Halophila ovalis* leaves exposed to marine heatwaves (control vs. heatwave) in terms of: (a) epiphyte biomass and (b) per cent cover of epiphytes. Asterisks represent level of significance (*p < .05, **p < .01).

Leaf epiphyte biomass and cover were also not significantly affected by the treatments for *H. beccarii* (Figure 4a,b; Table S1). However, dry weight biomass for the epiphytes attached to *H. ovalis* was significantly lower under the MHW treatment (an average of 0.0008 g compared to 0.0020 g, respectively; Figure 4a; Table S2; *F* = 7.85, *p* < .05) along with epiphyte percentage cover on the leaves (averages of 64.0% compared to 89.5%; Figure 4b; Table S2; *F* = 20.25, *p* < .01).

## 4 DISCUSSION

With climate change, there is an increase in the number, intensity and duration of MHW events (Frölicher et al., 2018). As a result, there is recorded global loss of seagrass, particularly loss of density, as well as subsequent changes to community structure (Smale et al., 2019). In areas of East and South‐East Asia, seagrass loss has been recorded in Vietnam (Luong et al., 2012), however, there are relatively few recordings of MHW effects on seagrasses in these tropical regions compared to records made in temperate regions (Bennett et al., 2022; Deguette et al., 2022; Fraser et al., 2014). The present study shows significant resistance of two seagrass species, *H. beccarii* and *H. ovalis* found in tropical and subtropical regions, to a simulated 10‐day spring MHW event. Notably, these species did have differing ecophysiological responses. While *H. ovalis* was unaffected by the MHW (although epiphyte biomass and cover declined), *H. beccarii* thrived under MHW conditions. That is, although the surface area of *H. beccarii* leaves present at the beginning of the heatwave decreased to a greater extent in the MHW treatment, possibly due to increased decomposition rates with higher temperatures (Kelaher et al., 2018; Trevathan‐Tackett et al., 2020), the greater production of new leaves in this treatment meant that ramets overall were resistant to the event. Therefore, this study revealed that spring MHWs may not be detrimental to seagrass survival and growth in Hong Kong.

Seagrasses, including *Halophila* spp., typically have high growth rates and fecundity which allow them to respond rapidly to environmental perturbations (Marbà & Duarte, 1998; O'Brien et al., 2018; Rasheed et al., 2008). *Halophila ovalis* has an approximate plastochron interval of 4.4 days for each pair of leaves (Erftemeijer & Stapel, 1999; Vermaat et al., 1995), while for *H. baccarii* plastochron interval has not been defined but new shoots can be formed within 2–3 days (Le Xuan et al., 2022). In this study, the production of new leaves for *H. ovalis* was similar under the different temperature treatments and corresponded to the rates of the known plastochron interval for this species. In contrast, *H. beccarii* increased its leaf turnover rate by boosting the number of new leaves grown under the MHW condition compared to ramets grown under the control condition. Similar enhancement is also seen in other seagrass species under simulated warming, such as increased metabolism and leaf production rate (Egea et al., 2019; Liu et al., 2022). The MHW condition in this study was 27°C, which likely falls within the thermal tolerance range of these species: while *H. beccarii*'s thermal optimal range has not been formally recorded, that of *H. ovalis* is recorded to be between 25 and 30°C (Ralph, 1998), and subtropical and tropical seagrasses typically have a thermal optimal range up to 35°C (Campbell et al., 2006; Ralph, 1998). For the genus *Halophila* and other small, fast‐growing species, there is a characteristic tendency for higher above‐ground biomass production in comparison to below‐ground (Duarte & Chiscano, 1999). As the above‐ground seagrass sections can contribute to photosynthesis and higher productivity, increasing below‐ground biomass may become a respiratory burden under increased temperature (Lee et al., 2007). Therefore, by allocating more resources to leaf production under MHW conditions, *H. beccarii* can exploit the increased temperature which is within its tolerance range by boosting photosynthetic potential.

Rapid responses of seagrasses to environmental perturbations can include accelerated rhizome elongation, which can allow for fast recolonisation (*H. beccarii*; Fong, 2000). While not significantly affected by the MHW treatment in this study, *H. beccarii* and *H. ovalis* increased their rhizome length over the duration of the experiment, with a trend for this to occur more quickly under MHW conditions in both species. This result suggests fast recovery potential of the species to MHWs and other extreme events. Such quick recovery has already been observed in Hong Kong. For example, in summer 2017, a typhoon event removed *H. ovalis* seagrasses from Tung Chung Bay (the collection site for this study), however, the beginning of recovery was observed in spring 2018, by the presence and increased quantity of seedlings (Wong, 2018). Moreover, our survey of the site during collection in March 2022 indicates that the area cover of the seagrass in spring of this year was approximately the same as before the extreme event (14,000–16,000 m^2^). Similar increases in other small, colonising seagrass species, such as *H. ovalis*, have also been seen elsewhere (e.g. in Australia after a MHW event; Kendrick et al., 2019). Such recovery capacity—even under MHW events—will be important given the ecological functions of seagrasses, such helping to support coastal biodiversity and habitat complexity (Ranjitham et al., 2008).

The results from this study also indicate that increased temperature from a MHW can affect the root length of *H. beccarii*, which was lower under the MHW treatment. Reduced root length can be a self‐thinning mechanism of the seagrass in response to high seagrass density (Jiang et al., 2020). Although the seagrass in the experimental tanks was not overcrowded, there was higher above‐ground biomass of *H. beccarii* under the MHW treatment by the end of the experiment, which may have generated this response in root length. There are various negative consequences of restricted root length, such as decreased resistance to hydrodynamic exposure and reduced capacity to absorb nutrients (Jiang et al., 2020). However, lowered root length can also shorten the path length for oxygen supply (Hovey et al., 2012; Jiang et al., 2019). This change can be beneficial to the seagrass, particularly as the oxygen requirement for respiration is higher under elevated temperatures (Rasmusson et al., 2020). Therefore, in response to MHWs, shorter root length may be advantageous and facilitate increased respiration rates.

Changes in epiphyte cover can have important implications for both the seagrass it occurs on and the species which consume it. Here, we identified that *H. ovalis* had lower biomass and percentage cover of epiphytes on its leaves under the MHW treatment. Lower epiphyte biomass can be beneficial to the seagrass as it improves access to light, consequently leading to better health of the seagrass (Brodersen et al., 2015) and increasing seagrass productivity (Orth & Van Montfrans, 1984). In contrast, the epiphytes that grow on the seagrass leaves are also a considerable food source for mesograzers. For example, several gastropod species have been recorded grazing on the epiphytes of *H. ovalis* (Fong et al., 2018). Seagrass‐epiphyte‐grazer relationships have been studied rigorously in the past 40 years (Duffy et al., 2001; Orth & Van Montfrans, 1984; van Montfrans et al., 1984), and show that loss of epiphytes on seagrass leaves can affect the grazing species and translate to changes on many trophic levels (Bendell, 2009). Our results indicate a lower epiphytic abundance on *H. ovalis* leaves which may negatively impact herbivorous invertebrates in Hong Kong seagrass meadows.

While the results of this study demonstrate the encouraging positive and neutral responses of the two species to short MHWs, longer MHWs events can increase the severity of deleterious physiological responses from seagrasses (Olsen et al., 2012; Pruckner et al., 2022), particularly for events when the ambient condition is warmer (i.e. summer MHWs; Bass et al., 2023). Moreover, stressors co‐occurring with MHWs can lead to more deleterious effects, such as changes in nutrient levels and turbidity, which can have much larger repercussions on the seagrass ecosystems (Kendrick et al., 2019; Orth et al., 2006). In large coastal cities, such as Hong Kong, land reclamation, pollution and harmful algal blooms threaten coastal habitat loss and fragmentation (Lai et al., 2016). These disturbances, along with extreme climatic events, such as tropical cyclones (Fong, 2000) and MHWs of increased severity and duration have to potential to compound and exacerbate the currently observed patterns of global seagrass loss.

## 5 CONCLUSION

This study indicates that short MHWs in springtime pose no significant additional threat to the survival or growth of these two seagrass species. Importantly, their differing responses, and the responses of the epiphytes which grow on them, may have bottom‐up influences on the biotic environment of the habitats. The ongoing presence of these seagrasses will have an important role in shaping the associated floral and faunal biodiversity, and reduction of further biodiversity loss. Therefore, by understanding more about the responses of different seagrasses to potential environmental threats, conservation strategies can be adjusted to their individual habitat requirements.

## AUTHOR CONTRIBUTIONS

**Alissa V. Bass:** Conceptualization (equal); data curation (lead); formal analysis (lead); investigation (lead); methodology (equal); visualization (lead); writing – original draft (lead); writing – review and editing (equal). **Laura J. Falkenberg:** Conceptualization (equal); funding acquisition (lead); methodology (equal); project administration (equal); supervision (lead); writing – review and editing (equal).

## FUNDING INFORMATION

Alissa V Bass is supported by the Hong Kong PhD Fellowship Scheme (HKPFS) established by the Research Grants Council (RGC) of Hong Kong.

## Supporting information


Figure S1.

Table S1.
Table S2.

## Data Availability

The data that support the findings of this study can be accessed at: https://doi.org/10.48668/T2WBX8.
