# Supplementary material for: Two tropical seagrass species show differing indicators of resistance to a marine heatwave
Source: Ecol Evol. 2023 Jul 14;13(7):e10304. doi: 10.1002/ece3.10304 (PMC10345732; doi:10.1002/ece3.10304)
Supplement: Supplementary file 1 — Figure S1. [file ECE3-13-e10304-s001.docx]

**Two tropical seagrass species show differing indicators of resistance to a marine heatwave**

Alissa V. Bass, Laura J. Falkenberg

*Supplementary Information*

Figure S1: Schematic diagram of the experimental system. Sand filtered seawater was continuously fed into each of the header tanks. This water was then maintained at either ambient temperature with a chiller or MHW temperature with aquarium bar heaters. This water was then pumped into the relevant seagrass tanks (*n* = 10 per temperature). Water was then able to drain from the seagrass tanks into the drainage system.
