# Supplementary material for: Two tropical seagrass species show differing indicators of resistance to a marine heatwave
Source: Ecol Evol. 2023 Jul 14;13(7):e10304. doi: 10.1002/ece3.10304 (PMC10345732; doi:10.1002/ece3.10304)
Supplement: Supplementary file 2 — Table S1. Table S2. [file ECE3-13-e10304-s002.docx]

**Differing responses of two tropical seagrasses to a simulated marine heatwave**

Alissa V. Bass, Laura J. Falkenberg

*Supplementary Information*

|  | | | | |
| --- | --- | --- | --- | --- |
| Response variable | df | F | P | Residuals (df) |
| Rhizome biomass | 1 | 1.012 | 0.344 | 7.93 |
| Rhizome length | 1 | 4.15 | 0.077 | 7.81 |
| Root biomass | 1 | 0.16 | 0.686 | 7.75 |
| Longest root length | 1 | 6.4 | **0.036*** | 7.79 |
| Total leaf biomass | 1 | 1.084 | 0.329 | 7.86 |
| Leaf SA change | 1 | 13.441 | **0.008**** | 6.83 |
| Largest new leaf SA^ | 1 | - | 0.12 | - |
| Leaf number change | 1 | 7.89 | **0.0232*** | 7.91 |
| Number new leaves | 1 | 6.14 | **0.0039**** | 7.91 |
| Loss of old leaves | 1 | 0.490 | 0.504 | 7.75 |
| Epiphyte biomass^ | 1 | - | 0.490 | - |
| Epiphyte percentage | 1 | 0.2707 | 0.6177 | 7.58 |

Table S1: Results of one-way ANOVAs examining the responses of *H. beccarii* to marine heatwaves (control vs. heatwave). Bold values indicate statistical significance (critical level of *p* < 0.05), and asterisks indicate level of significance*.* ^Results for *H. beccarii* largest new leaf SA for *H. beccarrii* were analysed with non-parametric Kruskal-wallis chi-squared test, as data was non-normal. Epiphyte biomass was analysed using a zero-inflated beta distribution regression model and then ANOVA type III performed.

|  | | | | |
| --- | --- | --- | --- | --- |
| Response variable | df | F | P | Residuals (df) |
| Rhizome biomass | 1 | 0.339 | 0.577 | 7.85 |
| Rhizome length | 1 | 0.260 | 0.62 | 7.86 |
| Root biomass | 1 | 0.118 | 0.74 | 7.86 |
| Longest root length | 1 | 0.651 | 07.95 | 0.44 |
| Total leaf biomass | 1 | 0.260 | 0.624 | 7.95 |
| Leaf SA change | 1 | 0.011 | 0.9197 | 7.1 |
| Largest new leaf SA | 1 | 0.254 | 0.628 | 7.96 |
| Leaf number change | 1 | 0.141 | 0.718 | 7.86 |
| Number new leaves | 1 | 0.0071 | 0.935 | 7.94 |
| Loss of old leaves | 1 | 0.1966 | 0.669 | 7.86 |
| Epiphyte biomass | 1 | 7.85 | **0.023*** | 7.97 |
| Epiphyte percentage | 1 | 20.25 | **0.002**** | 7.47 |

Table S2: One-way ANOVA for *H. ovalis* comparing responses to control vs MHW treatments. Bold values indicate statistical significance (critical level of *p* < 0.05), and asterisks indicate level of significance*.*
